# Therapeutic effects of Kangxian Yanshen formula on patients with chronic kidney disease stages 3–4: a retrospective cohort study

**DOI:** 10.3389/fmed.2024.1450561

**Published:** 2024-09-24

**Authors:** Aojiao Chu, Wenqian Wei, Ni Liu, Fan Zhang, Xianwen Zhang, Xueling Li, Rong Zheng, Zhifang Ma, Yi Li, Shu Rong, Yifei Zhong

**Affiliations:** ^1^Department of Nephrology A, Longhua Hospital Shanghai University of Traditional Chinese Medicine, Shanghai, China; ^2^Department of Nephrology, Shanghai General Hospital, Shanghai, China; ^3^Shanghai Fengxian District Hospital of Traditional Chinese Medicine, Shanghai, China

**Keywords:** Kangxian Yanshen formula, chronic kidney disease, eGFR, retrospective study, Chinese medicine

## Abstract

**Background:**

This study retrospectively evaluated the actual efficacy of Kangxian Yanshen Formula Chinese medicine on renal function-related indicators in chronic kidney disease (CKD) stage 3–4 patients.

**Methods:**

In this retrospective cohort study, we collected 212 adult CKD patients with baseline estimated glomerular filtration rate (eGFR) of 15–60 ml/min/1.73 m^2^. All participants received usual care (i.e., Western medications), and participants in the exposure group (*n* = 109) were additionally prescribed Kangxian Yanshen Formula Chinese medicine. The primary outcome was an adjusted hazard risk and 95% confidence interval (95% CI) of a 30% decrease in eGFR at month 36 from baseline.

**Results:**

In terms of eGFR, among participants treated with additional Kangxian Yanshen Formula, after adjusting for covariates, there was a 57.1% reduction in the risk of a 30% decline from baseline in eGFR among participants in the Kangxian Yanshen Formula group compared with the Western medicine group (adjusted hazard risk: 0.429; 95% CI 0.269–0.682). In addition, participants in the Kangxian Yanshen Formula group had a significantly higher change in eGFR from baseline to month 12 than those in the western medicine group (3.40 ± 11.62 versus −3.87 ± 8.39; between-group difference Δ5.61 [± 2.26 standard deviation] mL/min/1.73 m^2^; *P* = 0.014). Participants in both groups showed a decreasing trend in eGFR at months 24 and 36.

**Conclusion:**

In patients with stage 3–4 CKD, Kangxian Yanshen Formula Chinese medicine therapy may help delay eGFR decline, but high-quality randomized controlled trials are needed to validate the results further.

## Graphical Abstract

The figure summarizes the retrospective cohort study on the therapeutic effects of Kangxian Yanshen Formula in patients with chronic kidney disease stages 3–4. The study background, participants, primary outcome, and conclusion are presented. The primary outcome graph shows a 57.1% reduction in the risk of a 30% decline from baseline eGFR among participants in the Kangxian Yanshen Formula group compared to the Western medicine group. 
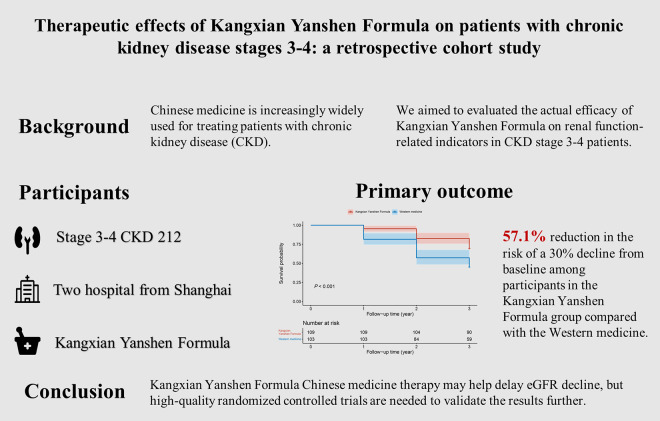

## 1 Introduction

With the increasing number of people with diabetes, high blood pressure, obesity, and the accelerating process of population aging, chronic kidney disease (CKD) has emerged as one of the most prominent causes of overall mortality and mortality from noncommunicable diseases worldwide (1, 2). Approximately 700 million people are estimated to be affected by CKD, and the prevalence rate is about 9.1%, among which China has the largest number of patients (approximately 130 million) (2–4). CKD is a progressive condition characterized by structural and functional changes to the kidney, which occur due to multifarious etiologies (5, 6). Symptoms of CKD are usually insidious. There are a variety of severe complications in advanced CKD, such as progressive uremia, volume overload, electrolyte abnormalities, acidemia, anemia, mineral and bone disorders, and possibly death if left untreated (7–9). A CKD diagnosis means kidney failure is likely inevitable and frequently irreversible over time (10).

The current treatments include slowing the progression of renal function loss, targeting primary diseases such as diabetes and hypertension, and therapy for complications such as cardiovascular disease, anemia, mineral and bone disorder, hydroelectrolytic disorders, and metabolic acidosis, and as a last resort, preparation for kidney failure with replacement therapy (6–9). However, the mortality of patients on dialysis can be as high as 20% per year, and transplantation is limited by organ shortage and transplant rejection (10, 11). The high number of affected individuals and the significant adverse impact of CKD should prompt enhanced efforts for better prevention and treatment (6–9). In recent years, many large clinical studies have confirmed that sodium-dependent glucose transporters 2 (SGLT-2) inhibitors, represented by dapagliflozin, have a clear cardio-renal benefit while lowering glucose potently (12–14), and clinical guidelines have recommended it as the first-line drug for CKD patients (15). However, SGLT-2 inhibitors are also accompanied by numerous adverse effects.

In China and many other Asian countries, traditional Chinese medicine is often used as an alternative strategy for CKD (16–18). The biological activities and therapeutic effects of some traditional Chinese medicines in CKD have been reported in the previous publications (19–22). Studies, including in review well-designed randomized controlled trials and meta-analyses, have suggested that taking Chinese medicines as adjunctive therapy to conventional medications may be beneficial for patients with CKD (23). Kangxian Yanshen Formula is an effective formula summarized by our research department under traditional Chinese medicine theory and long-term clinical practice. This study was a retrospective analysis of real-world data to observe the therapeutic effects of Kangxian Yanshen Formula-based traditional Chinese medicine on patients with stage 3–4 CKD.

## 2 Materials and methods

This retrospective cohort study was conducted in the nephrology clinics of Longhua Hospital Shanghai University of Traditional Chinese Medicine and Shanghai General Hospital in Shanghai. This study was approved by the Ethics Committee of Longhua Hospital Shanghai University of Traditional Chinese Medicine (No: 2022LCSY073), exempting informed consent because there was minimal risk for the retrospective cohort participants.

### 2.1 Study population

We retrospectively collected participants who were regularly followed up in the nephrology clinic from January 2012 to September 2023 at two hospitals via electronic case review. Inclusion criteria: 1) patients with stage 3–4 CKD as judged by the Kidney Disease: Improving Global Outcomes (KDIGO) guidelines (24); 2) aged between 18 and 80 years; 3) having received western medication and/or herbal prescriptions based on Kangxian Yanshen Formula; and 4) undergoing regular follow-up at the outpatient clinics of the two centers.

### 2.2 Kangxian Yanshen formula

All participants received routine CKD management according to the KDIGO guidelines, using Western medications to control blood pressure, glycosylated hemoglobin, and lipids and monitoring proteinuria (25). Participants in the exposed group were then supplemented with a Kangxian Yanshen Formula Chinese medicine, which is an empirical formula used in Longhua Hospital for treating chronic renal insufficiency, which has been clinically applied for decades and has good clinical efficacy, with the effect of benefiting qi, tonifying kidney and activating blood circulation. The formula mainly consisted of Astragali radix (Huang Qi, tonifying qi), Chuanxiong (Chuanxiong Rhizoma, activating blood circulation), *Curcuma zedoaria* (Christm, activating blood circulation), Cicada flower (tonifying kidneys), and *Mantidis ootheca* (Sangpiaoxiao, tonifying kidneys). In the exposure group, participants received regular follow-up visits from an outpatient physician to take the Kangxian Yanahen Formula Chinese medicine.

### 2.3 Data collection

This study retrospectively collected participants’ baseline demographic characteristics and laboratory parameters at four time points (baseline, 12th, 24th, and 36th months), including age, gender, receipt of Immunization/hormone therapy, use of angiotensin-converting enzyme inhibitor (ACEI)/angiotensin receptor blocker (ARB) medications, serum creatinine (umol/L), serum uric acid (umol/L), 24-hour proteinuria (g/d), and estimated glomerular filtration rate (eGFR, mL/min/1.73 m^2^) based on Chronic Kidney Disease Epidemiology Collaboration creatinine equation (26).

### 2.4 Outcomes

The primary outcome of this study was eGFR. Our primary parameter of interest was the surrogate endpoint of a 30% decrease in eGFR from baseline (27). Secondary outcomes were absolute changes from baseline in eGFR, 24-hour proteinuria, serum creatinine, and serum uric acid at 36 months.

### 2.5 Statistical analysis

Continuous variables are expressed as mean ± standard deviation (SD), and categorical variables are described as absolute frequency (n) and relative frequency (%). For primary outcome analyses, we first developed Cox proportional hazards models to estimate the adjusted hazard ratio (HR) and 95% confidence interval (95% CI) for a 30% decrease in eGFR from baseline in the Kangxian Yanshen Formula group relative to the Western medicine group. Three Cox regression models were fitted in total. Model 1 was not adjusted. Model 2 was adjusted for age and gender. The complete model (model 3) was adjusted for age, gender, immunization/hormone therapy, use of ACEI/ARB or not, baseline serum creatinine, eGFR, serum uric acid, and 24-hour proteinuria. Next, we performed subgroup analyses of Cox proportional hazards models for age (< 65 or ≥ 65 years), gender (male or female), immunization/hormone therapy (yes or not), use of ACEI/ARB (yes or not), and baseline CKD staging. For secondary outcome analyses, *t*-tests were used to compare the between- and within-group differences between the two groups of patients to investigate the effect of treatment at different time periods. Statistical analyses were performed using SPSS (IBM SPSS Statistics for Windows, Version 26.0; IBM Corp.). All analyses were statistically significant at *P* < 0.05.

## 3 Result

This study retrospectively collected 212 CKD patients with stage 3–4 CKD at baseline. The clinical characteristics are shown in Table 1. The mean age of participants was 60.5 ± 14.1. There were 50.5% of participants who were male (107). In addition, a few participants received immunization/hormone therapy (46, 21.7%). There were 148 (69.8%) participants with CKD stage 3 and 64 (30.2%) with CKD stage 4. Of these, 109 were continuously treated with Kangxian Yanshen Formula Chinese medicine, and 103 were treated with Western medicine as a control.

**TABLE 1:** Characteristics of included participants (*n* = 212).

	Total (212)	Kangxian Yanshen formula (*n* = 109)	Western medicine (*n* = 103)	*P*-value
Gender				0.054 ^1^
Male	107 (50.5%)	48 (44.0%)	59 (57.3%)	
Female	105 (49.5%)	61 (56.0%)	44 (42.7%)	
Age, years				0.820 ^1^
< 65	128 (60.4%)	65 (59.6%)	63 (61.2%)	
> = 65	84 (39.6%)	44 (40.4%)	40 (38.8%)	
Immunization/hormone therapy				0.011 ^1^
No	166 (78.3%)	93 (85.3%)	73 (70.9%)	
Yes	46 (21.7%)	16 (14.7%)	30 (29.1%)	
Use of ACEI/ARB or not				0.033 ^1^
No	92 (43.4%)	55 (50.5%)	37 (35.9%)	
Yes	120 (56.6%)	54 (49.5%)	66 (64.1%)	
CKD stage				0.220 ^1^
Stage 3	148 (69.8%)	72 (66.1%)	76 (73.8%)	
Stage 4	64 (30.2%)	37 (33.9%)	27 (26.2%)	
eGFR, mL/min/1.73 m^2^	40 (28, 50)	40 (27, 48)	41 (30, 50)	0.622 ^2^
24-h proteinuria, g/d	0.65 (0.19, 1.22)	0.57 (0.08, 1.11)	0.70 (0.31, 1.43)	0.028 ^2^
Serum creatinine, umol/L	145 (118, 185)	142 (113, 187)	149 (121, 180)	0.535 ^2^
Serum uric acid, umol/L	433 (364, 487)	432 (391, 483)	433 (355, 504)	0.622 ^2^

Data were presented as number (%) and median (interquartile range). CKD, chronic kidney disease; ACEI, angiotensin-converting enzyme inhibitor; ARB, angiotensin receptor blocker; eGFR, estimated glomerular filtration rate.

^1^ Pearson’s Chi-squared test.

^2^ Wilcoxon rank sum test.

### 3.1 Primary outcomes

**FIGURE 1:**
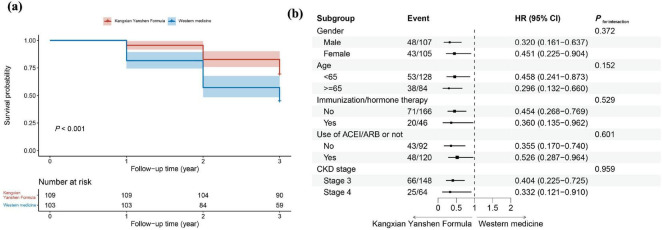
**(a)** survival analysis graphs for participants in both groups. The *x*-axis represents the follow-up time, while the *y*-axis shows survival probability. The red line represents the Kangxian Yanshen Formula group, and the blue line represents the Western group. **(b)** Subgroup analysis for Kangxian Yanshen Formula versus Western medicine. Models are adjusted for age, gender, immunization/hormone therapy, use of ACEI/ARB or not, baseline eGFR, serum creatinine, serum uric acid, 24-h proteinuria, and CKD stage, except the subgroup variable itself. HR, hazard ratio; ACEI, angiotensin-converting enzyme inhibitor; ARB, angiotensin receptor blocker; CKD, chronic kidney disease; eGFR, estimated glomerular filtration rate.

A Cox regression model was fitted with whether an individual had a 30% decrease in eGFR from baseline at the 36th month as the dependent variable and group as the independent variable. The results showed that, after adjusting for age, gender, immunization/hormone therapy, use of ACEI/ARB or not, baseline eGFR, serum creatinine, serum uric acid, 24-hour proteinuria, and CKD stage, participants in the Kangxian Yanshen Formula group had a 57.1% (adjusted HR: 0.429; 95% CI 0.269–0.682) lower risk of having a 30% decrease in eGFR from baseline compared to the Western medicine group (Table 2). Survival curves of the two groups are shown in Figure 1a. The results were robust in subgroup analyses and remained unchanged regarding magnitude and significance (Figure 1b).

**TABLE 2:** Kangxian Yanshen Formula and the risk of a 30% decline in eGFR from baseline in CKD patients.

	Model 1 (HR, 95% CI)	Model 2 (HR, 95% CI)	Model 3 (HR, 95% CI)
Group			
Western medicine	Ref.	Ref.	Ref.
Kangxian Yanshen Formula	0.512 (95% CI 0.333–0.787)	0.489 (95% CI 0.317–0.757)	0.429 (95% CI 0.269–0.682)

Model 1: Unadjusted. Model 2: Adjusted age and gender. Model 3: Adjusted age, gender, immunization/hormone therapy, use of ACEI/ARB or not, baseline eGFR, serum creatinine, serum uric acid, 24-hour proteinuria, and CKD stage. HR, hazard ratio; ACEI, angiotensin-converting enzyme inhibitor; ARB, angiotensin receptor blocker; CKD, chronic kidney disease; eGFR, estimated glomerular filtration rate.

### 3.2 Secondary outcomes

#### 3.2.1 eGFR

**FIGURE 2:**
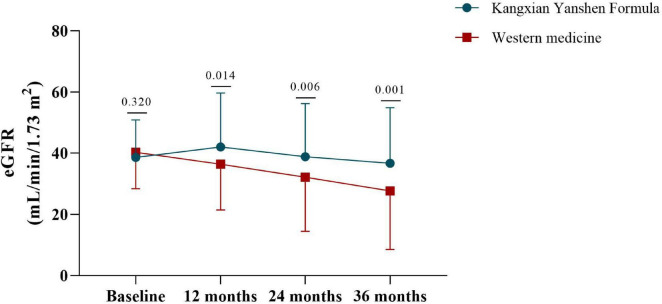
Line graphs depicting the changes in eGFR over time in both groups of participants. The *x*-axis represents the time points of measurement, while the *y*-axis shows the eGFR values in mL/min/1.73m^2^. The blue line represents the Kangxian Yanshen Formula group, and the red line represents the Western group. Error bars indicate standard deviation. Differences between groups at specific time points are marked with *P*-value.

**FIGURE 3:**
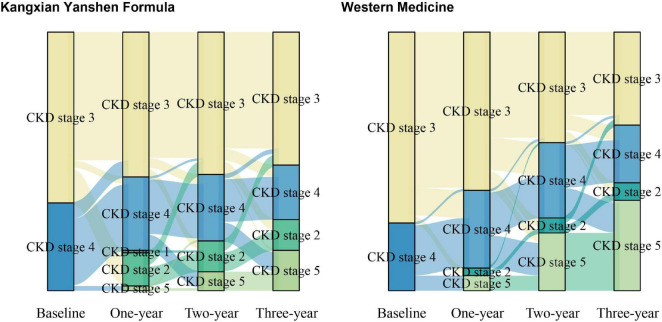
Stacked bar charts illustrating the progression of CKD staging over time for participants in both groups. The *x*-axis shows the time points of assessment, while the *y*-axis represents the percentage of participants in each CKD stage. Different colors are used to represent the various CKD stages (Stage 1: dark green, Stage 2: green, Stage 3: yellow, Stage 4: blue, Stage 5: line green). The left panel shows data for the Kangxian Yanshen Formula group, and the right panel shows data for the Western group.

Figure 2 shows the absolute change in eGFR stratified by treatment group. Participants in the Kangxian Yanshen Formula group had a significantly higher magnitude of change in eGFR from baseline to month 12 than those in the Western medicine group (3.40 ± 11.62 versus −3.87 ± 8.39; between-group difference Δ5.61 [± 2.26 SD] mL/min/1.73 m^2^; *P* = 0.014). A decreasing trend was observed at months 24 and 36, and changes in the magnitude of the eGFR difference were most pronounced at month 36 (Kangxian Yanshen Formula group: Δ−1.90 [± 12.49 SD] mL/min/1.73 m^2^ versus Western medicine group: Δ−12.61 [± 14.63 SD] mL/min/1.73 m^2^; between-group difference Δ9.05 [± 2.56 SD] mL/min/1.73 m^2^) (Figure 2 and Table 3). As shown in Figure 3, the Sankey diagram shows a change in CKD staging over time in both groups of participants based on eGFR assessment.

**TABLE 3:** Within-group changes in renal function-related indices in both groups of participants.

	Kangxian Yanshen Formula (*n* = 109)	*P*_for within–group_	Western medicine (n = 103)	*P*_for within–group_
**eGFR, 1.73 mL/min/1.73 m^2^**				
Baseline	38.55 ± 12.36	–	40.21 ± 11.90	–
Change at month 12	3.40 ± 11.62	0.003	−3.87 ± 8.39	< 0.001
Change at month 24	0.23 ± 11.66	0.836	−8.11 ± 12.03	< 0.001
Change at month 36	−1.90 ± 12.49	0.116	−12.61 ± 14.63	< 0.001
**24-h proteinuria, g/d**				
Baseline	0.83 ± 0.91	–	1.08 ± 1.08	–
Change at month 12	0.12 ± 0.78	0.118	0.25 ± 1.31	0.055
Change at month 24	0.21 ± 0.92	0.020	0.41 ± 1.59	0.011
Change at month 36	0.51 ± 1.18	< 0.001	0.48 ± 1.59	0.003
**Serum creatinine, umol/L**				
Baseline	155.28 ± 51.9		158.24 ± 52.07	
Change at month 12	−2.18 ± 43.27	0.601	34.84 ± 92.53	< 0.001
Change at month 24	13.21 ± 60.9	0.026	95.86 ± 201.07	< 0.001
Change at month 36	32.1 ± 81.96	< 0.001	193.96 ± 301.63	< 0.001
**Uric acid, umol/L**				
Baseline	435.67 ± 84.42		428.44 ± 124.1	
Change at month 12	18.92 ± 287.19	0.493	−4.72 ± 124.79	0.702
Change at month 24	−5.61 ± 84.5	0.490	−13.54 ± 129.54	0.291
Change at month 36	−31.63 ± 104.41	0.002	−24.7 ± 142.99	0.083

eGFR, estimated glomerular filtration rate.

#### 3.2.2 24-h proteinuria

**FIGURE 4:**
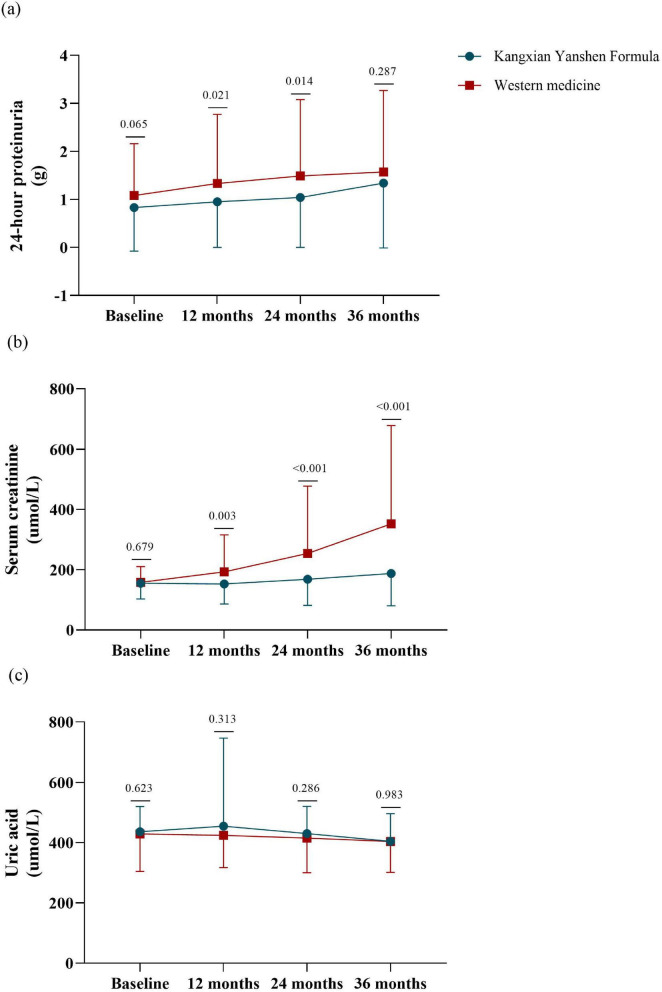
Line graphs depicting the changes in **(a)** 24-h proteinuria; **(b)** serum creatinine; **(c)** uric acid over time in both groups of participants. The x-axis represents the time points of measurement, while the y-axis shows the outcomes. The blue line represents the Kangxian Yanshen Formula group, and the red line represents the Western group. Error bars indicate standard deviation. Differences between groups at specific time points are marked with *P*-value.

Figure 4a shows the absolute change in 24-hour proteinuria stratified by the treatment group. The magnitude of change in 24-hour proteinuria from baseline to month 12 was significantly higher among participants in the Kangxian Yanshen Formula group than in the Western medicine group (0.12 ± 0.78 versus 0.25 ± 1.31; between-group difference Δ−0.39 [± 0.17 SD] g/d; *P* = 0.021). All showed an increasing trend at months 24 and 36, with the magnitude of change in the 24-hour proteinuria difference being most pronounced at month 24 (Kangxian Yanshen Formula group: Δ0.21 [± 0.92 SD] g/d versus Western medicine group: Δ0.41 [± 1.59 SD] g/d; between-group difference Δ−0.45 g/d; *P* = 0.021). Group difference Δ−0.45 [± 0.18 SD] g/d; *P* = 0.014) (Figure 4a and Table 3).

#### 3.2.3 Serum creatinine

Figure 4b shows the absolute change in serum creatinine stratified by the treatment group. Serum creatinine remained almost constant among participants in the Kangxian Yanshen Formula group, whereas participants in the Western medicine group showed a trend toward a significant increase in serum creatinine. The magnitude of change of serum creatinine difference was most pronounced at month 36 (Kangxian Yanshen Formula group: Δ32.1 [± 81.96 SD] umol/L versus Western medicine group: Δ193.96 [± 301.63 SD] umol/L; between-group difference Δ−164.83 [± 33.76 SD] umol/L; *P* < 0.001) (Figure 4b and Table 3).

#### 3.2.4 Serum uric acid

Figure 4c shows the absolute change in serum uric acid stratified by treatment group. There was a slight downward trend in serum uric acid for participants in both groups. The magnitude of change in serum uric acid difference was most pronounced at month 12 but was not statistically significant (Kangxian Yanshen Formula group: Δ18.92 [± 287.19 SD] umol/L versus Western medicine group: Δ−4.72 [± 124.79 SD] umol/L; between-group difference Δ30.87 [± 30.51 SD] umol/L; *P* = 0.313) (Figure 4c and Table 3).

## 4 Discussion

In this two-center, retrospective study, we confirmed the renoprotective effect of Kangxian Yanshen Formula in patients with CKD stage 3–4. Kangxian Yanshen Formula was significantly associated with a delayed deterioration of renal function over 36 months (HR: 0.429; 95% CI 0.269–0.682) compared with conventional Western medicine. This protective effect was more pronounced in the first 12 months, as evidenced by a slight increase in eGFR and a non-significant rise in 24-hour proteinuria. Inevitably, although participants in both groups subsequently experienced a decrease in eGFR, the decline was smaller in the Kangxian Yanshen Formula group than in the Western medicine group. These data demonstrate the advantages and feasibility of Kangxian Yanshen Formula over Western medicine alone for treating CKD.

In recent years, an increasing number of clinical trials and animal studies have confirmed that some herbal compounds can delay the progression of CKD based on Chinese medicine theories and their molecular mechanisms (28–34). Lin et al. (35) explored the relationship between using prescribed herbal medicines and the risk of end-stage renal disease in CKD patients by searching the National Health Insurance Study database in Taiwan from 2000 to 2005. After adjusting for confounding variables, the risk of end-stage renal disease was significantly reduced by 60% in participants who used herbal medicines compared to those who did not (cause-specific hazard ratio 0.41, 95% CI 0.37–0.46). Further analyses showed that patients using wind dampness-dispelling or harmonizing formulas had a lower risk of end-stage renal disease, whereas patients using dampness-dispelling and purgative formulas had an increased risk of end-stage renal disease. This finding suggests that specific herbal medicines are associated with the progression of renal function. As a comparison, this study found that consumption of Kangxian Yanshen Formula was associated with a 57.1% reduction in the risk of a 30% decrease in participant eGFR from baseline, like the results of Lin et al. (35).

Our previous review (20) reported that traditional Chinese medicine has anti-inflammatory, antioxidant, antifibrotic, and immunomodulatory effects in treating CKD as an alternative therapy to immunosuppressive or anti-inflammatory drugs; however, its long-term effects remain unproven. As a complement, the current retrospective study demonstrated a role in adding Kangxian Yanshen Formula treatment during a 3-year follow-up period. The non-significant difference in proteinuria changes may be attributed to 1) the high intra-individual biological variability in 24-hour proteinuria and the relatively small sample size; 2) participants in both groups received standard treatment with Western medicine in the early stages of CKD, and Kangxian Yanshen Formula did not show additional proteinuria-reducing effects after entering stages 3–4; 3) the renoprotective effect of Kangxian Yanshen Formula may be independent of reducing proteinuria.

For delayed degradation of eGFR, several randomized controlled trials in the past reported similar findings. Mao et al. (36) recruited 567 patients with stage 4 CKD; participants in the treatment group received the Bupi Yishen Formula, while the control group took Losartan. The difference in eGFR slope between both groups over a 48-week interval was −2.24 mL/min/1.73 m^2^ (95% CI: −4.01, −0.46), and no between-group differences in adverse events were observed. Chan et al. (37) published a study based on Rehmannia-6-Based Chinese Medicine treatment comparing Standard care in patients with diabetic nephropathy stage 2-3 showed that the slopes of eGFR change were per 1.73 m^2^ −2.0 (95% CI: −0.1, −3.9) and −4.7 (95% CI: −2.9, −6.5) ml/min, in other words, the difference between both groups was −2.7 ml/min/1.73 m^2^ (95% CI: −5.3, −0.1). In comparison, the intergroup difference in eGFR at week 48 in this study was −10.71 mL/min/1.73 m^2^ (95% CI: −14.41, −7.02), with an advantage of being based on a real-world population, and the long follow-up period.

It is well known that renal function declines with age, especially in elderly patients (38). The maximum urine osmolality of the elderly decreased by about 20% compared to younger patients (39). In the subgroup analysis, we found that Kangxian Yanshen Formula had a protective effect on renal function in both middle-aged and older adults, and it was better in the latter. It means that Kangxian Yanshen Formula may help mitigate renal function decline in elderly patients and is a good choice for treating elderly CKD patients.

With advances in complex technologies (e.g., mass spectrometry), many active compounds of traditional medicines have been identified, and the efficacy of traditional Chinese medicine in CKD has been confirmed (20). For example, Radix Astragali (Huangqi) is essential to the Kangxian Yanshen Formula. Studies have shown that the active components in Radix Astragali have antioxidant properties, which can scavenge free radicals in the body and reduce damage to the kidneys caused by oxidative stress, which helps to maintain renal function (40); in addition, Radix Astragali has an inhibitory effect on tissue fibrogenesis (41), which may help prevent renal fibrosis and its induced functional impairment.

Unfortunately, no adverse events related to Chinese medicines were recorded in this study, but reports of nephrotoxicity of Chinese medicines are not rare and should not be ignored (42). Although Kangxian Yanshen Formula reported beneficial effects on renal function in this study, using Chinese herbal medicines should be evaluated and guided by consulting a medical professional in practical applications, especially for end-stage renal disease.

## 5 Strength and limitations

The strength of this study is that it explored the effect of Kangxian Yanshen Formula on the long-term impact on disease progression in patients with CKD stages 3–4 from different renal function-related indicators. Nevertheless, as a retrospective study, some limitations are unavoidable. First, this study was not a randomized sample, and sample selection may be affected by selective bias because the investigator cannot control what happened in the past and may not be able to comprehensively include all relevant data, thus affecting the representativeness of the study results. Second, eGFR calculation based on serum creatinine may be affected by body weight and diet, and data availability made us lack adjustment for the body weight variable. Third, there was a lack of sufficient sensitivity analyses (e.g., intention-to-treat analysis) to confirm our results’ robustness. Fourth, limitations of the study design prevented excluding other unknown factors or confounding variables from influencing the results, leading to an inability to draw clear causal inferences. Fifth, during data collection, we recorded drug side effects, but due to the long follow-up period, none of the patients were able to recall the adverse events that occurred, and we were not able to collect serious side effects, such as liver damage, acute renal failure, and electrolyte abnormalities. Sixth, using a fixed herbal formula for treating diseases, including CKD, during the follow-up period is likely to be unreasonable in practice (43, 44); therefore, there may be some bias in adding the therapeutic effect of Kangxian Yanshen Formula. Another limitation of this study is the lack of detailed information on Western medicine treatments. This information could provide valuable context for understanding the overall treatment regimen and potential interactions with the Kangxian Yanshen Formula.

## 6 Conclusion

In conclusion, this study provides preliminary evidence that Kangxian Yanshen Formula can be used as an additional therapeutic strategy for patients with stage 3−4 CKD, as it significantly delays the deterioration of renal function. However, this conclusion was limited by the study design, and further studies, including randomized controlled trials with larger samples and pharmacological studies, are needed regarding the renoprotective effects of Kangxian Yanshen Formula.

## Data Availability

The original contributions presented in the study are included in the article/supplementary material, further inquiries can be directed to the corresponding authors.

## References

[1] Kalantar-Zadeh K Jafar T Nitsch D Neuen B Perkovic V. Chronic kidney disease. Lancet. (2021) 398:786–802. 10.1016/S0140-673600519-5 34175022

[2] Matsushita K Ballew S Wang A Kalyesubula R Schaeffner E Agarwal R. Epidemiology and risk of cardiovascular disease in populations with chronic kidney disease. *Nat Rev Nephrol.* (2022) 18:696–707. 10.1038/s41581-022-00616-6 36104509

[3] Rashid I Katravath P Tiwari P D’Cruz S Jaswal S Sahu G. Hyperuricemia–a serious complication among patients with chronic kidney disease: A systematic review and meta-analysis. *Explorat Med.* (2022) 3:249–59. 10.37349/emed.2022.00089 39280247

[4] Glassock R Warnock D Delanaye P. The global burden of chronic kidney disease: Estimates, variability and pitfalls. *Nat Rev Nephrol.* (2017) 13:104–14. 27941934 10.1038/nrneph.2016.163

[5] Huang R Fu P Ma L. Kidney fibrosis: From mechanisms to therapeutic medicines. *Signal Transduct Target Ther.* (2023) 8:129. 10.1038/s41392-023-01379-7 36932062 PMC10023808

[6] Kalantar-Zadeh K Li P. Strategies to prevent kidney disease and its progression. *Nat Rev Nephrol.* (2020) 16:129–30. 10.1038/s41581-020-0253-1 32005966

[7] Ruiz-Ortega M Rayego-Mateos S Lamas S Ortiz A Rodrigues-Diez R. Targeting the progression of chronic kidney disease. *Nat Rev Nephrol.* (2020) 16:269–88. 10.1038/s41581-019-0248-y 32060481

[8] Stenvinkel P Painer J Kuro O Lanaspa M Arnold W Ruf T Novel treatment strategies for chronic kidney disease: Insights from the animal kingdom. *Nat Rev Nephrol.* (2018) 14:265–84. 10.1038/nrneph.2017.169 29332935

[9] Ravid J Kamel M Chitalia V. Uraemic solutes as therapeutic targets in CKD-associated cardiovascular disease. *Nat Rev Nephrol.* (2021) 17:402–16. 10.1038/s41581-021-00408-4 33758363

[10] Zoccali C Mark P Sarafidis P Agarwal R Adamczak M Bueno de Oliveira R Diagnosis of cardiovascular disease in patients with chronic kidney disease. *Nat Rev Nephrol.* (2023) 19:733–46. 10.1038/s41581-023-00747-4 37612381

[11] Breyer M Susztak K. The next generation of therapeutics for chronic kidney disease. *Nat Rev Drug Discov.* (2016) 15:568–88. 10.1038/nrd.2016.67 27230798 PMC5511522

[12] Heerspink H Stefánsson B Correa-Rotter R Chertow G Greene T Hou F Dapagliflozin in patients with chronic kidney disease. *N Engl J Med.* (2020) 383:1436–46. 10.1056/NEJMoa2024816 32970396

[13] McMurray J Solomon S Inzucchi S Køber L Kosiborod M Martinez F Dapagliflozin in patients with heart failure and reduced ejection fraction. *N Engl J Med.* (2019) 381:1995–2008. 10.1056/NEJMoa1911303 31535829

[14] Wiviott S Raz I Bonaca M Mosenzon O Kato E Cahn A Dapagliflozin and cardiovascular outcomes in type 2 diabetes. *N Engl J Med.* (2019) 380:347–57. 10.1056/NEJMoa1812389 30415602

[15] Roddick A Wonnacott A Webb D Watt A Watson M Staplin N UK kidney association clinical practice guideline: Sodium-glucose co-transporter-2 (SGLT-2) inhibition in adults with kidney disease 2023 UPDATE. *BMC Nephrol.* (2023) 24:310. 10.1186/s12882-023-03339-3 37880609 PMC10598949

[16] Newman D Cragg G. Natural products as sources of new drugs over the nearly four decades from 01/1981 to 09/2019. *J Nat Prod.* (2020) 83:770–803. 10.1021/acs.jnatprod.9b01285 32162523

[17] Khaled A Ahmed E Mamdouh M Saad H Mohamed A Sobhy M Natural angiotensin converting enzyme inhibitors: A safeguard against hypertension, respiratory distress syndrome, and chronic kidney diseases. *Phytother Res.* (2023) 37:5464–72. 10.1002/ptr.7987 37675925

[18] Arabi S Bahari H Hamidipor S Bahrami L Feizy Z Nematy M The effects of curcumin-containing supplements on inflammatory biomarkers in hemodialysis patients: A systematic review and meta-analysis. *Phytother Res.* (2022) 36:4361–70. 10.1002/ptr.7642 36205586

[19] Zhong Y Deng Y Chen Y Chuang P Cijiang He J. Therapeutic use of traditional Chinese herbal medications for chronic kidney diseases. *Kidney Int.* (2013) 84:1108–18. 10.1038/ki.2013.276 23868014 PMC3812398

[20] Zhong Y Menon M Deng Y Chen Y He J. Recent advances in traditional chinese medicine for kidney disease. *Am J Kidney Dis.* (2015) 66:513–22. 10.1053/j.ajkd.2015.04.013 26015275

[21] Zou T Liu Z Cao P Zheng S Guo W Wang T Fisetin treatment alleviates kidney injury in mice with diabetes-exacerbated atherosclerosis through inhibiting CD36/fibrosis pathway. *Acta Pharmacol Sin.* (2023) 44:2065–74. 10.1038/s41401-023-01106-6 37225845 PMC10545759

[22] Lin P Qiu F Wu M Xu L Huang D Wang C Salvianolic acid B attenuates tubulointerstitial fibrosis by inhibiting EZH2 to regulate the PTEN/Akt pathway. *Pharm Biol.* (2023) 61:23–9. 10.1080/13880209.2022.2148169 36524761 PMC9762854

[23] Zhao M Yu Y Wang R Chang M Ma S Qu H Mechanisms and efficacy of Chinese herbal medicines in chronic kidney disease. *Front Pharmacol.* (2020) 11:619201. 10.3389/fphar.2020.619201 33854427 PMC8039908

[24] National Kidney Foundation. K/DOQI clinical practice guidelines for chronic kidney disease: Evaluation, classification, and stratification. *Am J Kidney Dis.* (2002) 39:S1–266. 11904577

[25] Levin A Stevens P. Summary of KDIGO 2012 CKD guideline: Behind the scenes, need for guidance, and a framework for moving forward. *Kidney Int.* (2014) 85:49–61. 10.1038/ki.2013.444 24284513

[26] Inker L Schmid C Tighiouart H Eckfeldt J Feldman H Greene T Estimating glomerular filtration rate from serum creatinine and cystatin C. *N Engl J Med.* (2012) 367:20–9. 10.1056/NEJMoa1114248 22762315 PMC4398023

[27] Thompson A Lawrence J Stockbridge N. GFR decline as an end point in trials of CKD: A viewpoint from the FDA. *Am J Kidney Dis.* (2014) 64:836–7. 10.1053/j.ajkd.2014.09.006 25446026

[28] Tan Y Li R Zhou P Li N Xu W Zhou X Huobahuagen tablet improves renal function in diabetic kidney disease: A real-world retrospective cohort study. *Front Endocrinol.* (2023) 14:1166880. 10.3389/fendo.2023.1166880 37404303 PMC10315672

[29] Sun M Ye H Zheng C Jin Z Yuan Y Weng H. Astragalin ameliorates renal injury in diabetic mice by modulating mitochondrial quality control via AMPK-dependent PGC1α pathway. *Acta Pharmacol Sin.* (2023) 44:1676–86. 10.1038/s41401-023-01064-z 36859596 PMC10374896

[30] Ren Q Tao S Guo F Wang B Yang L Ma L Natural flavonol fisetin attenuated hyperuricemic nephropathy via inhibiting IL-6/JAK2/STAT3 and TGF-β/SMAD3 signaling. *Phytomedicine.* (2021) 87:153552. 10.1016/j.phymed.2021.153552 33994251

[31] Zhou W Chen M Liu H Si Z Wu W Jiang H Dihydroartemisinin suppresses renal fibrosis in mice by inhibiting DNA-methyltransferase 1 and increasing Klotho. *Acta Pharmacol Sin.* (2022) 43:2609–23. 10.1038/s41401-022-00898-3 35347248 PMC9525601

[32] Wang R Yuan W Li L Lu F Zhang L Gong H Resveratrol ameliorates muscle atrophy in chronic kidney disease via the axis of SIRT1/FoxO1. *Phytother Res.* (2022) 36:3265–75. 10.1002/ptr.7499 35606908

[33] Wang B Yang L Yang L Liang Y Guo F Fu P Fisetin ameliorates fibrotic kidney disease in mice via inhibiting ACSL4-mediated tubular ferroptosis. *Acta Pharmacol Sin.* (2024) 45:150–65. 10.1038/s41401-023-01156-w 37696989 PMC10770410

[34] Han X Zhang J Zhou L Wei J Tu Y Shi Q Sclareol ameliorates hyperglycemia-induced renal injury through inhibiting the MAPK/NF-κB signaling pathway. *Phytother Res.* (2022) 36:2511–23. 10.1002/ptr.7465 35434887

[35] Lin M Chiu Y Chang J Lin H Lee C Chiu G Association of prescribed Chinese herbal medicine use with risk of end-stage renal disease in patients with chronic kidney disease. *Kidney Int.* (2015) 88:1365–73. 10.1038/ki.2015.226 26244923

[36] Mao W Yang N Zhang L Li C Wu Y Ouyang W Bupi Yishen formula versus losartan for non-diabetic stage 4 chronic kidney disease: A randomized controlled trial. *Front Pharmacol.* (2020) 11:627185. 10.3389/fphar.2020.627185 33708125 PMC7941267

[37] Chan K Kwong A Tan K Lui S Chan G Ip T Add-on rehmannia-6-based Chinese medicine in type 2 diabetes and CKD: A multicenter randomized controlled trial. *Clin J Am Soc Nephrol.* (2023) 18:1163–74. 10.2215/CJN.0000000000000199 37307005 PMC10564374

[38] Schaeffner E Ebert N Delanaye P Frei U Gaedeke J Jakob O Two novel equations to estimate kidney function in persons aged 70 years or older. *Ann Intern Med.* (2012) 157:471–81. 10.7326/0003-4819-157-7-201210020-00003 23027318

[39] Rowe J Shock N DeFronzo R. The influence of age on the renal response to water deprivation in man. *Nephron.* (1976) 17:270–8. 10.1159/000180731 951013

[40] Peng A Gu Y Lin S. Herbal treatment for renal diseases. *Ann Acad Med Singap.* (2005) 34:44–51. 15726219

[41] Zhu Y Chai Y Xiao G Liu Y Xie X Xiao W Astragalus and its formulas as a therapeutic option for fibrotic diseases: Pharmacology and mechanisms. *Front Pharmacol.* (2022) 13:1040350. 10.3389/fphar.2022.1040350 36408254 PMC9669388

[42] Yang B Xie Y Guo M Rosner M Yang H Ronco C. Nephrotoxicity and Chinese herbal medicine. *Clin J Am Soc Nephrol.* (2018) 13:1605–11. 10.2215/CJN.11571017 29615394 PMC6218812

[43] Wu P Li J Yan H Guo R Lv Y Wang Y. Status and prospect of international standardization of TCM diagnosis. *Pharmacol Res.* (2021) 171:105746. 10.1016/j.phrs.2021.105746 34186191

[44] Gobe G Shen K. Chinese herbal medicines and chronic kidney disease: A positive outcome in a large patient study in Taiwan. *Kidney Int.* (2015) 88:1223–6. 10.1038/ki.2015.300 26649659

